# Hernia: Its Etiology, Symptoms, and Treatment

**Published:** 1901-12

**Authors:** 


**Hernia: its Etiology, Symptoms, and Treatment.** By W. McAdam Eccles, M.S. (Lond.). Pp. viii., 231. London: Baillière, Tindall & Cox. 1900.

In this manual, as the author states in the preface, no attempt has been made to deal exhaustively with any division of the subject of hernia, and nothing new is to be found in it. But as a clear and concise description of the various forms of hernia, their diagnosis and treatment, the book deserves praise. We would specially single out for praise the chapters dealing with the application of trusses. This branch of the subject is dealt with in proportionately greater detail than are some of the others, *e.g.* the so-called radical operative treatment. In fact, we know of no better short account of the treatment of the various forms of hernia with trusses than that given here, an account which it will well repay the majority of practitioners to carefully study. The numerous excellent illustrations in the book constitute a special feature. It is somewhat disappointing to find the operative treatment of hernia not more fully discussed. The book gives the operating surgeon but little help in the selection of a method, and but little information as to the results achieved by modern operations for the radical cure of hernia.

